# Computational approaches to lipid-based nucleic acid delivery systems

**DOI:** 10.1140/epje/s10189-023-00385-5

**Published:** 2023-12-14

**Authors:** Giovanni Settanni

**Affiliations:** 1 Faculty of Physics and Astronomy, Ruhr University Bochum, Universitätstrasse 150, 44801 Bochum, Germany; 2 Department of Physics, Johannes-Gutenberg University Mainz, Staudingerweg 7, 55099 Mainz, Germany

## Abstract

Nucleic acid-based therapies have shown enormous effectiveness as vaccines against the recent COVID19 pandemics and hold great promises in the fight of a broad spectrum of diseases ranging from viral infections to cancer up to genetically transmitted pathologies. Due to their highly degradable polyanionic nature, nucleic acids need to be packed in sophisticate delivery vehicles which compact them up, protect them from early degradation and help delivery them to the right tissue/cells. Lipid-based nanoparticles (LNP) represent, at present, the main solution for nucleic acid delivery. They are made of a mixture of lipids whose key ingredient is an ionizable cationic lipid. Indeed, the interactions between the polyanionic nucleic acids and the ionizable cationic lipids, and their pH-dependent regulation in the life cycle of the nanoparticle, from production to cargo delivery, mostly determine the effectiveness of the therapeutic approach. Notwithstanding the large improvements in the delivery efficiency of LNPs in the last two decades, it is estimated that only a small fraction of the cargo is actually delivered, stimulating further research for the design of more effective LNP formulations. A rationally driven design would profit from the knowledge of the precise molecular structure of these materials, which is however still either missing or characterized by poor spatial resolution. Computational approaches have often been used as a molecular microscope either to enrich the available experimental data and provide a molecular-level picture of the LNPs or even simulate specific processes involving the formation and/or the molecular mechanisms of action of the LNP. Here, I review the recent literature in the field.

## Introduction

Nucleic acid therapeutics include several classes of drugs which have a nucleic acid as active ingredient. The nucleic acid, when reaching the target cell, may help either silencing a gene (in the case of small interfering RNA or siRNA) or expressing a specific protein (in the case of messenger RNA or mRNA) [1, 2]. Given the potentially very broad applicability of the technique and its high specificity, many research efforts have been dedicated to dissect and improve it, and the successes of the COVID-19 mRNA-based vaccination campaign [3, 4] have provided an early demonstration of the capabilities of the approach.

The direct injection of RNA, a labile poly-anion, is not a viable solution to its delivery, as the molecule is quickly degraded by the immune system of the treated organism. Thus, a large variety of delivery systems have been developed [5, 6]. Presently, the delivery vehicles of choice are lipid-based nanoparticles (LNP) [7]. Notwithstanding the successes, present-days delivery systems are thought to effectively deliver only a small fraction of their RNA cargo [8]. In addition only specific tissues can be targeted at the moment.

Thus, there is a need for the development of new delivery vehicles. A rational design of those nanoparticles is hindered by the lack of knowledge of the molecular structure and molecular mechanism of action of present-day solutions. Computer modeling and molecular dynamics simulations paired to experiments provide a powerful tool of investigation to get a molecular-level understanding of the involved phenomena. The aim of this mini review is to summarize recent results about LNP structure and mechanism of action obtained using computational methods.

LNPs are made of a mixture of lipids typically including a ionizable cationic lipid (IL) responsible for interacting with nucleic acids at low pH, various helper lipids, including phospholipids and cholesterol, thought to help with LNP stabilization, and a small molar fraction of PEGylated lipids which help sizing the nanoparticles, and prevent their aggregation and early degradation upon injection [7, 9]. It is worth noting that this composition is the result of several decades of efforts in optimizing the lipid formulations for nucleic acid delivery [10], which among other things, led to replace permanent cationic lipids with ionizable cationic lipids. The former, although functional *in vitro*, are associated with high toxicity *in vivo*, unlike the latter, which are neutral under physiological pH [11].

LNPs are usually made in microfluidics devices by rapid mixing a solution containing the RNA at low pH with an ethanol-solubilized lipid formulation [12]. The electrostatically driven interactions between the positively charged IL component and the negatively charged RNA, as well as the interactions between all the other components, drive the formation of the nanoparticles.

The molar fraction of each component as well as the chemical structure of the IL and helper lipids determines size and internal structure of the LNP, as well as its pK$_a$. The latter parameter is thought to play an important role in delivery efficiency, and values between 6.2 and 6.5 have been shown to be optimal [13] (at least under specific conditions). The ratio between the number of ionizable amine groups on the lipids and the phosphate groups on the RNA backbone is an important parameter indicated with N/P ratio. The size of LNPs is comprised in a range from several tens to few hundred nanometers. After production, the LNPs are usually brought to a neutral or physiological pH for application or storage.

**Fig. 1:**
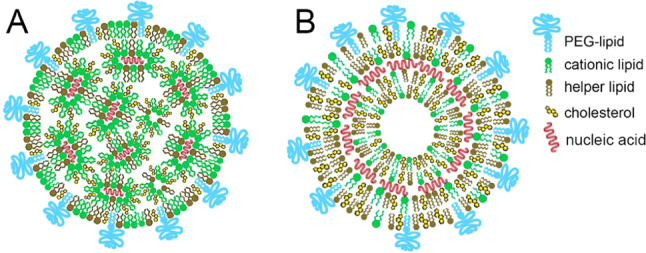
Simplified sketches of LNPs with either a dense core with disordered inverted hexagonal or inverted micellar structure (**A**) or a multi-lamellar structure (**B**). Figure adapted with permission from Ref. [18] Copyright ©2021 Tenchov et al. under license CC BY

Cryo-TEM imaging and SAXS/SANS experiments have shown that LNPs built as above may have a dense core, possibly rich in neutral IL and cholesterol [14], but also, in some case, a multi-lamellar structure [15–17], possibly indicating the formation of stacks of lipid bilayers intercalated by RNA-rich hydrophilic regions (Fig. 1). The PEG chains attached to the PEGylated lipids are thought to form an outer shell around the LNP.

**Fig. 2:**
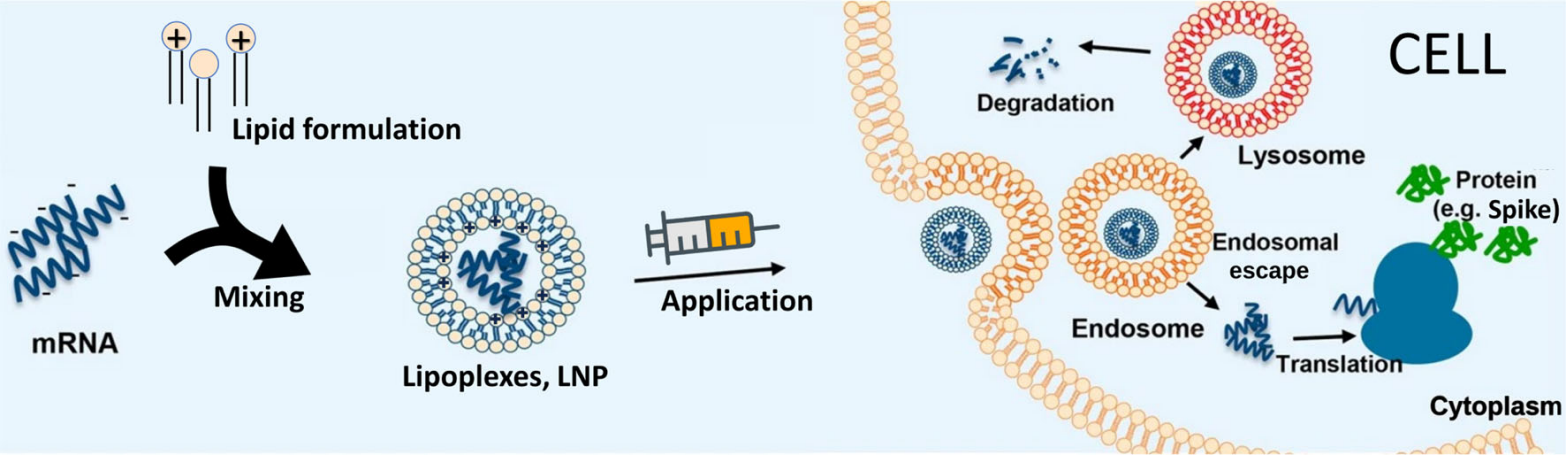
Simplified view of the production of lipid-based delivery systems for mRNA delivery and their mechanism of action. Figure adapted from Ref. [21] Copyright ©2020, HZG, CC BY. Please note that for the sake of simplicity many details relative to, for example, the pegylation of the nanoparticles or the presence of a large variety of structures (channels, receptors, etc.) on the cell membrane and inside the cell have been omitted

Upon injection, the size of the LNP and the presence of PEG chains on its surface reduce early disposal from the immune system and let the nanoparticle reach the target cells. Along the way, cleavage of the PEG chains is thought to occur [19]. Cell entry occurs mainly via endocytosis, although other mechanisms have also been observed [20]. Inside the endosome, the LNP is exposed to an increasingly acidic pH which quickly reaches below the pK$_a$ value of the nanoparticle. This process is thought to drive a conformational change in the LNP due to the protonation of the ILs. The exact nature of this change is the subject of intense investigation. As a matter of fact, while most of the LNPs proceed to the lysosome and are degraded, a fraction of them manage to deliver their RNA cargo through the endosomal membrane (endosomal escape) into the cytoplasm, where it will be able to interact with the cell machinery and produce the required therapeutic effect (Fig. 2).

**Fig. 3:**
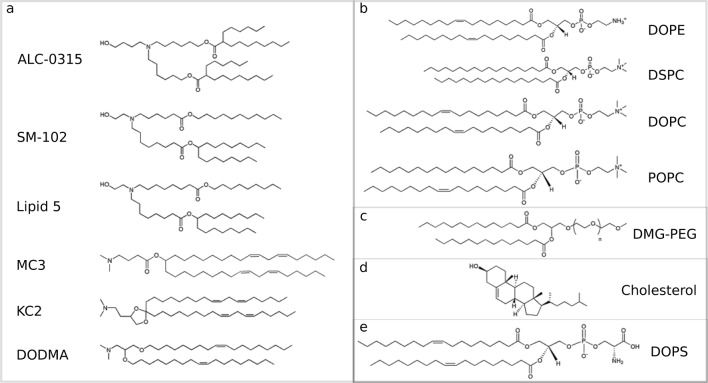
The chemical structure of some of the lipids discussed in the review, including ionizable cationic lipids (**a**), helper phospholipids (**b**), an example of PEG lipid (**c**), cholesterol (**d**), an example of anionic lipid present in the endosomal membrane (**e**)

In the following sections I consider several computational approaches that have been proposed recently in the literature to help and characterize structural and mechanistic aspects of the LNPs. Some of the chemical components of the systems, including IL and helper lipids, are reported in Fig. 3.

## LNP internal structure, lipid distribution and pH-dependent behavior

**Fig. 4:**
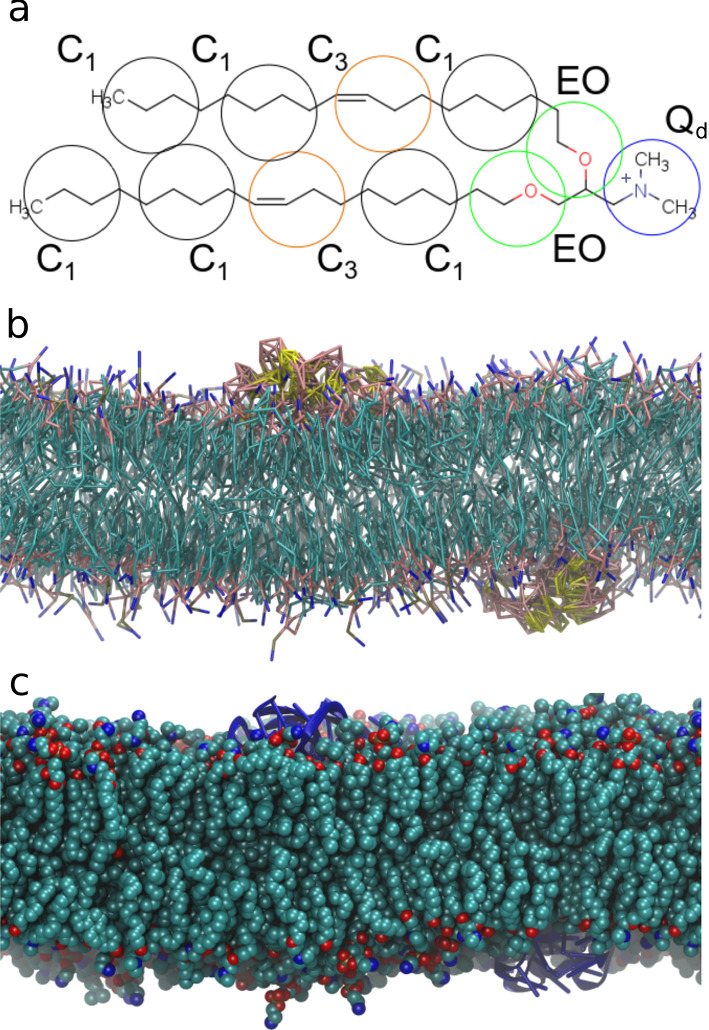
A coarse-grained representation of a IL (DODMA) is superimposed to its atomistic structure. Each coarse-grained interacting bead (circles) represent groups of several atoms (**a**). A lipid bilayer with an adsorbed RNA chain (yellow-pink or dark blue) in coarse-grained (**b**) and atomistic (**c**) representation. Water and ions are omitted for clarity. Figure from Ref. [22] copyright©2021 Settanni et al. under license CC BY-NC-ND

As mentioned above, LNPs are produced at low pH (typically pH 4.0), where ILs are charged and later brought to physiological conditions (pH 7.4) where ILs can become neutral. Simulations have been used to try and understand the characteristics of the lipid formulation under those conditions, including, in some case, their interactions with the RNA. The complete self-assembly of the LNP, given the relatively large length scales involved, has been considered only rarely (see next section) and most often using coarse-grained representations (Fig. 4). On the other hand, the local molecular structure inside the nanoparticle can also be investigated on a smaller scale involving few hundred lipids typically pre-assembled in a bilayer configuration.

Early work from Khalid et al. [23] analyzed the self-assembly of short segments of double-stranded DNA (dsDNA) with zwitterionic and cationic lipids using a coarse-grained representation [24]. Subsequent work from Corsi et al. [25] demonstrated with a similar approach the possibility of observing the lamellar to inverted hexagonal phase transition in lipid mixtures containing also cationic lipids and dsDNA.

**Fig. 5:**
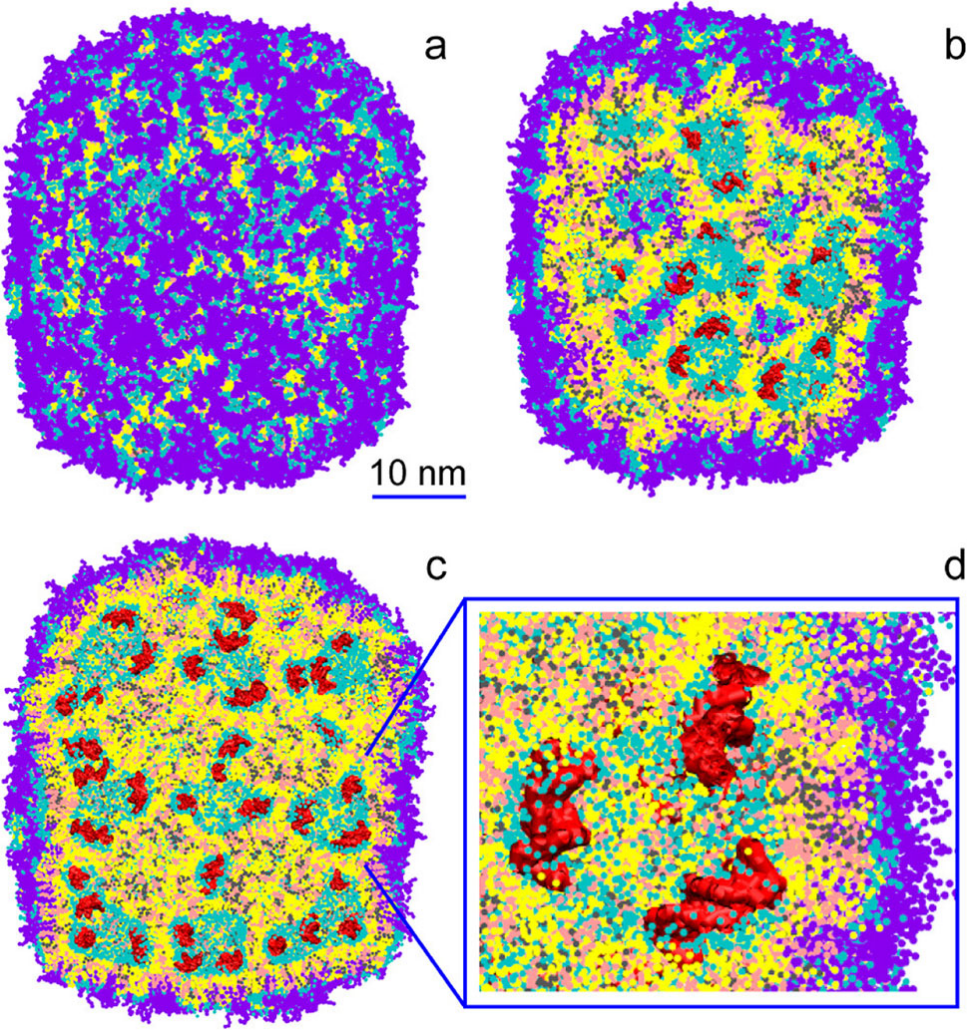
The LNP as obtained in Ref. [26]. Full view (**a**), cross sections (**b**, **c**) and a magnification of the inner structure (**d**) are shown. IL (yellow), cholesterol (pink), DSPC (gray), polar lipid groups (cyan), PEG-lipids (violet) and nucleic acid (red) are shown, water is omitted. Figure adapted with permission from Ref. [26] copyright©2012 the American Chemical Society

In one comprehensive work on LNPs, which appeared in combination with experiments, Leung et al. [26] studied the structure of a nanoparticle containing the ionizable cationic lipid 2,2-dilinoleyl-4-(2-dimethylaminoethyl)-[1,3]-dioxolane (DLinKC2-DMA or KC2) as well as the phospholipid 1,2-distearoyl-sn-glycero-3-phosphocholine (DSPC), cholesterol and the PEGylated lipid N-[(methoxy polyethylene glycol 2000 carbamyl]-1,2-dimyristyloxlpropyl-3-amine (PEG-c-DMA) in the 4/1/4/1 molar ratio. Segments of siRNA with an N/P ratio of about 4 were also present in the simulations. By means of a coarse-grained approach based on the MARTINI force field [27–29], the authors explored the self-assembly of the core of the nanoparticle as a function of the hydration level. They found that at low hydration level, the protonated KC2 molecules form inverted micelles by surrounding water-filled compartments containing short segments of siRNA, with the siRNA adsorbing to the internal surface of the inverted micelles. This arrangement was resembling closely a disordered hexagonal phase (Fig. 5).

Gindy et al. [30] investigated the role of the helper phospholipid in the stabilization of LNPs by means of molecular dynamics simulations. The authors used the OPLS united atom force field [31, 32]. The systems that they studied included the protonated IL Lipid 3 (dimethyl[(2R)-1-[(10E,13E)-nonadeca-10,13-dien-1-yloxy]-3-(octyloxy)propan-2-yl]amine), a highly transfecting IL identified in an earlier experimental screening [33]. The formulation included also cholesterol, PEG-lipids and one of three different phospholipids 1,2-dimyristoyl-sn-glycero-3-phosphocholine (DMPC), dilauroylphosphatidylcholine (DLPC) and DSPC, which have same head but tails of different length. The formulations were set up as bilayers, and molecular dynamics simulations of up to 90ns were performed for each system. The data showed that the stiffer bilayers, giving rise to sharper distributions of the chemical groups along the normal to the bilayers, as well as large tail order parameters for the phospholipids included DMPC, which has a tail of intermediate length with respect to the other tested phospholipids. The authors showed that the observed behavior is due to the presence of Lipid 3, an IL with 2 asymmetric tails, one short and one long. DMPC is the phospholipid which better helps to fill the volume gap in the bilayer originated by the short tail of Lipid 3. A stiffer bilayer was associated by the authors to possibly more stable NPs, with less demanding storage requirements.

Ramezanpour et al. [34] simulated mixtures containing the IL KC2 and the phospholipid 1-palmitoyl-2-oleoyl-sn-glycero-3-phosphocholine (POPC), as well as, in some case, cholesterol, at different pH levels, temperature and mixing ratios from 10 to 30%mol KC2. The atomistic simulations obtained with the CHARMM36 [35–37] force field, after validation with NMR data for KC2, contained 200 lipids in total and were set up in an initial bilayer configuration. They showed, along 700ns trajectories, that neutral KC2 accumulated in an oily phase between the two leaflets made mostly of POPC or a mixture of POPC and cholesterol. Only a small fraction of cholesterol joined the oily phase. At low pH, instead, the protonated KC2 remained in the leaflet and contributed to increase the tail order parameters of the phospholipids. These data supported the experimental hypothesis of LNPs with a dense core composed, at least partly, of neutral KC2 [26, 38].

Ermilova and Swenson [39] simulated bilayers containing up to 15%mol of the IL (6Z,9Z,28Z,31Z)-heptatriacont-6,9,28,31-tetraene-19-yl 4-(dimethylamino) butanoate (DLin-MC3-DMA or MC3) in a matrix of either 1,2-Dioleoyl-sn-glycero-phosphoethanolamine (DOPE) or 1,2-dioleoyl-sn-glycero-3-phosphocholine (DOPC), in order to understand the role played by the helper lipid. The simulations were run using the atomistic SLipids force field [40] (the parameters for neutral MC3 were obtained in the same work). System sizes of 200 lipids were considered, and the simulations were run for 300ns after an equally long equilibration. Further parallel tempering metadynamics simulations were performed to measure the free energy of permeation of water through the membranes. At the largest simulated concentration of MC3 the authors observe aggregation of the IL. However, the MC3 lipids do not accumulate in the inter-leaflet space as seen in [34] for KC2. In the simulations, while the MC3 head tends to align along the normal to bilayer with the phosphate of phospholipids, MC3 tails occupy locations closer to the head region than the tails of the phospholipids. The authors noted that the formation of specific interactions between IL and helper lipids, as observed for DOPE, may change the diffusivity and permeation properties of the bilayer.

Park et al. [41] developed the force field parameters compatible with CHARMM36 [35–37] for a large set of ILs and made them available through the CHARMM-GUI [42–44] platform. With those parameters they tested systems similar to those simulated previously in Refs. [34] and [39], that is formulations based on KC2 and on MC3, respectively. They demonstrated that the accumulation of neutral KC2 lipids inside the bilayer of KC2/POPC mixtures is also observed with their force field, and a similar phase separation also occurs for neutral MC3, although to a lower extent than KC2, possibly due to interactions of the MC3 head with phospholipids’ glycerol groups. They also simulated additional systems containing a realistic LNP formulation, including cholesterol and PEG-lipids. They observed the formation of IL-dependent interactions between PEG oxygens and the cationic groups of protonated ILs.

Paloncyova et al. [45] investigated the behavior of the two ILs present in the COVID19 vaccine formulations from Pfizer-Biontech [4] and Moderna [3], that are ALC-0315 ((4-hydroxybutyl)azanediyl)bis(hexane-6,1-diyl)bis(2-hexyldecanoate) and SM-102 (heptadecan-9-yl 8-(2-hydroxyethyl)[6-oxo-6-(undecyloxy)hexyl]aminooctanoate), respectively. Using an atomistic level of resolution and the AMBER [46] force field, they collected trajectories of up to 500ns for several different systems investigating the behavior of ILs in both the neutral and the protonated state, as well as with and without RNA. The systems contained a maximum of 200 lipids and some included cholesterol and DSPC. They observed that while in the protonated state both ILs can form bilayers either as single component or in mixture with the helper lipids, in the neutral state those ILs lose this property and tend to form aggregated phases or to accumulate in the space between the leaflets of bilayers formed by the helper lipids. Self-assembly simulations of formulations containing the protonated ILs, cholesterol and DSPC resulted in phase separation between regions rich in DSPC and cholesterol and regions rich in ILs. DSPC and cholesterol form ordered bilayer-like structures, ILs tend to occupy curved regions like the border of nanodisks, or the knot regions of the cubic phase or they surround the pores in porated bilayers. In the simulations in the presence of RNA, the nucleic acid tends to stick on the heads of the charged ILs, thus remaining typically on the surface of the lipid complex. The presence of ILs in these structures has only minor effects on the structure of the RNA.

**Fig. 6:**
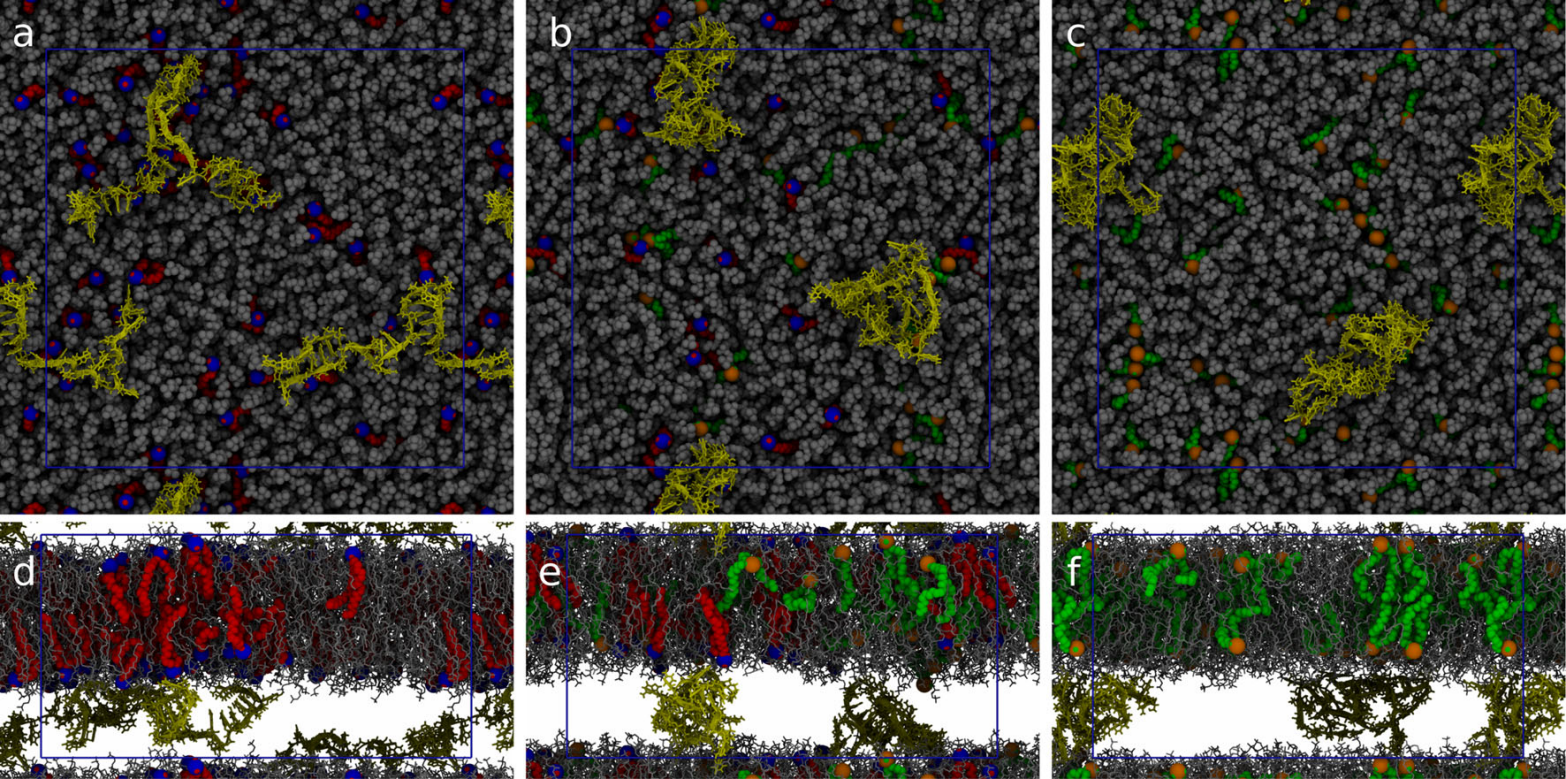
Top (**a**–**c**) and side (**d**–**f**) view of the systems described in Ref. [22]. Protonation fraction of 100%, 50% and 0% are reported in the left, middle and right panel, respectively. Protonated and neutral DODMA are rendered in red/blue and orange/green, respectively, DOPC in gray and RNA in yellow. Water and ions are omitted for clarity. Figure from Ref. [22] copyright©2021 Settanni et al. under license CC BY-NC-ND

In our recent work [22] we simulated the pH-dependent behavior of a formulation containing the IL DODMA (1,2-dioleyloxy-3-dimethylaminopropane) and the phospholipid DOPC (13/87 %mol) either with or without a random sequence of an mRNA 40mer. The systems were set up into a periodic bilayer to mimic the internal structure of a multi-lamellar lipid nanoparticle [16]. The systems contained 645 lipids in total and were simulated using a multiscale approach (Fig. 4): the simulations were initially run using the MARTINI coarse-grained force field for 10$\mu$s, to allow proper diffusion of the lipids. Then, after backmapping to an atomistic representation, the simulations were continued using the CHARMM36 [35–37] force field for another 1$\mu$s. The simulations revealed that, while an effective attractive interaction between RNA and IL is present at low pH (that is between the protonated form of the IL and the nucleotides), at high pH an effective repulsive interaction was observed. The neutral form of DODMA distributed itself closer to the bilayer center and showed a marked increase in leaflet flipping probability. However, possibly thanks to its small head, it did not aggregate in between the two leaflets as observed for other ILs (Fig. 6).

Trollmann and Böckmann [47] simulated the self-assembly of the lipid mixture contained in the Pfizer-Biontech COVID-19 vaccine [4] using multiple $\mu$s-long atomistic simulations (CHARMM36 [35–37] force field). They observed the formation of bilayer structures at low pH. An increase of pH, neutralizing the ILs, led to their separation in a oily phase in the space between the two leaflets, along with some cholesterol molecules, as observed for KC2 [34]. In the presence of mRNA, an increase in pH leaving partly protonated ILs led to the formation of inverted micelles of protonated ILs containing the RNA strands. The inverted micelles localized in the oily region formed by neutral ILs and cholesterol in between the two leaflets. DSPC could also partly contribute to the micelles; however, the most of it remained on the surface along with cholesterol and PEGlipids and formed a relatively ordered phase. Notably, a complete deprotonation of the ILs led to the expulsion of RNA to the solvent phase.

Cornebise et al. [48] simulated small micelles made of several different protonated ILs (obtained from an *in vivo* screening for transfection efficiency), in contact with an RNA fragment. The 1-$\mu$s-long simulations obtained with the OPLS3e force field revealed that the most effective ILs for transfection formed tendentially a larger number of interactions with the nucleobases than the other lipids, which instead interacted mostly with the RNA backbone. In particular, the most effective IL (Lipid 29, heptadecan-9-yl 8-[(3-[2-(methylamino)-3,4-dioxocyclobut-1-en-1-yl]aminopropyl)[8-oxo-8-(undecan-3-yloxy)octyl]amino]octanoate), which included a squaramide group, made $\pi$-stacking interactions with adjacent nucleobases. This trend is however not clear cut, and it could be specifically related to the class of ILs considered. A similar approach was undertaken by Rissanou et al. [49] who simulated, using the CHARMM36 force field [35–37], the complexation of RNA chains of different lengths with the ionizable lipid-like agent DML (2-[2-(acetyloxy)ethyl](4-[5-(4-[2-(acetyloxy)ethyl](2-[(9E,12E)-octadeca-9,12-dienoyloxy]ethyl)aminobutyl)-3,6-dioxopiperazin-2-yl]butyl)aminoethyl (9E,12E)-octadeca-9,12-dienoate) and collected information about the structure and the mechanism of assembly of the system.

Dehghani et al. [50] simulated at atomistic resolution lipid formulations containing the IL Lipid5 (heptadecan-9-yl 8-[(2-hydroxyethyl)[8-(nonyloxy)-8-oxooctyl]amino]octanoate), which resulted as a promising candidate from a previous experimental screening [51]. The authors analyzed the interplay between ILs and phospholipids in the formulation, by varying the relative concentration, while keeping the concentration of cholesterol constant to 40%mol. The system were set up in an initial bilayer configuration of area 12.5 nm$\times$12.5 nm and simulated for 3 $\mu$s. Half of the ILs were protonated, while the rest was neutral. The simulations show that when neutral ILs concentration exceeds a critical value (40%mol), they aggregate into a hydrophobic core forming between the two leaflets and containing also cholesterol molecules. A larger fraction of ILs helps decreasing the order of the lipid bilayer in terms of tail order parameters. In addition the protonated ILs tend to aggregate on the surface of the bilayer in high curvature regions as in correspondence to the hydrophobic core made of neutral IL. This phenomenon resembles what observed already in Ref. [45].

Molecules like phospholipids and cholesterol are common components of biological systems. The associated force field parameters, required to include those molecules in atomistic simulation, have been extracted with relatively good accuracy from a quite large amount of available data. On the contrary, simulation parameters for ILs are based on a smaller amount of data or obtained by analogy to similar molecules. It is thus important to test and validate those parameters and evaluate their ability to reproduce experimental data. Ibrahim et al. [52] took in consideration a very effective IL, MC3 [13]. They developed an atomistic force field for this IL using the standard protocol of the AMBER [46] force field and then compared it to the available force fields for MC3 obtained in the two studies presented above (the CHARMM36 force field [41] and the SLipids force fields [39]). They considered bilayers including neutral or protonated MC3 lipids at different molar fractions mixed with the phospholipid DOPC. The size of the systems ranged from 200 to 400 lipids, which were simulated for 600 ns each. The simulations were then compared with neutron reflectivity data. The study showed that the CHARMM and AMBER force field provided similar distributions for the protonated form of the IL, which remains in the bilayer along with the phospholipid. The neutral ILs, instead, tend to accumulate in between the two leaflets of the bilayer, resulting in an increased thickness of the bilayer (stronger in the AMBER than in the CHARMM force field), as observed in neutron reflectivity data. The SLipids force field, instead, showed an accumulation of the neutral form of the ILs on the external surface of the bilayer at the lipid–water interface resulting in a decreased thickness of the bilayer.

In a recent work, Ermilova and Swenson [53], using the SLipids force field as in their previous work [39] and parallel-tempered metadynamics, investigated the penetration capability of MC3, ALC-0315 and SM-102 in their neutral state, with respect to several different phospholipid membranes. They observed that the binding free energy of MC3 with different phospholipids correlated with their phase transition temperature and that ALC-0315 and SM-102 have a significantly larger affinity for the membrane than MC3, with a possible explanation being the different degree of saturation of the tails of the ILs.

## LNP self-assembly

In the rapid mixing of RNA solution and lipid formulations used for LNP preparation a self-assembly process occurs. Simulating this process in its entirety is challenging due to the large sizes involved. For this reason in many cases people reverted to use coarse-grained representations of the system or a step-wise building process, or both.

Leung et al. [26] in the same work mentioned earlier, after self-assembling the core of a KC2-based LNP into a disordered inverted-hexagonal phase around siRNA-rich water compartments, multiplied this core in all direction, coated it with phospholipids and PEG lipids, rehydrated the system, and simulated it for 10 $\mu$s. The resulting LNP was about 44nm in diameter and showed a similar disordered hexagonal phase as the initial building block (Fig. 5). The water compartments were about 3–9 nm in size, and overall a hydration of about 6 water molecules per lipid was measured in the NP. The PEG segments formed a protecting layer around the surface of the NP. The findings from the simulations were shown to reproduce very accurately experimental cryo-TEM, NMR and sucrose density gradient centrifugation data showing the presence of a nanostructured lipid core, as well as the complete encapsulation of the nucleic acid. The authors speculated that the emergence of the inverted micellar phase instead of the multi-lamellar phase observed in other studies could be related to the fraction of bilayer-forming lipids like DSPC in the formulation, which in this case was low.

Bruininks et al. [54, 55] used an approach similar to the one of Leung et al. [26]. Using the MARTINI force field [27, 56], they built the inner part of an LNP using a mixture of protonated DOTAP (1,2-dioleoyl-3-trimethylammonium-propane) lipids and phospholipids as well as fragments of dsDNA. In this way they obtained an inverted hexagonal phase around the DNA. Then they multiplied the core in several directions and coated it with a single leaflet made of the same lipid composition as the inner core and let the system relax. They used these LNPs to investigate the LNP mechanism of action, as it will be clarified in the next section.

**Fig. 7:**
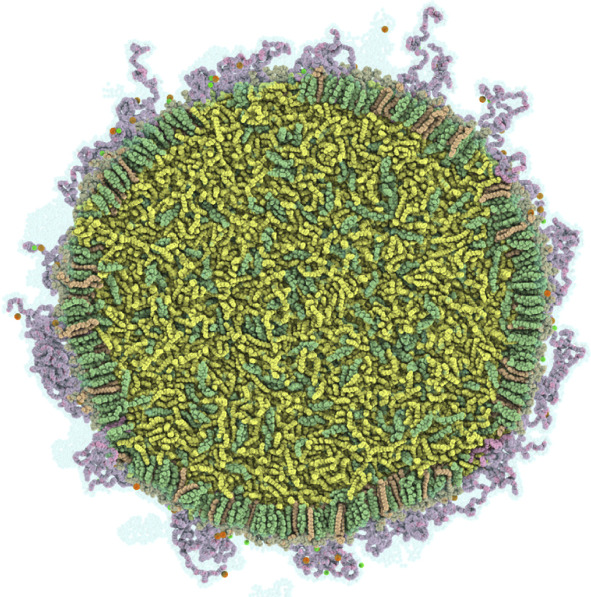
A cross section of the LNP as obtained in Ref. [47]. ALC-0315 (yellow), cholesterol (green), DSPC (orange), PEG-lipids (Violet) are reported. Figure from Ref. [47] copyright©2022 Biophysical Society under license CC BY-NC-ND 4.0 DEED

Trollmann and Böckmann [47], in the same work mentioned in the previous section, built a full RNA-free LNP based on the Pfizer-Biontech COVID19 vaccine [4] with a radius of 17.7 nm. Using an atomistic representation, they took as LNP core the oily phase rich in IL and cholesterol observed in the simulations of the formulation at high pH; then, they multiplied the core in several directions and coated it with an outer shell made of a single leaflet of DSPC, cholesterol and PEGylated lipid. $\mu$s-long equilibration of the system revealed that a small fraction of ILs moved to the surface of the LNP to be mostly buried by PEG chains, while the DSPC and cholesterol compacted into a leaflet with the same degree of order of pure DSPC/cholesterol mixtures (Fig. 7). They further used the electron density distribution inside the nanoparticle to compute the expected SAXS spectra and compare it with experimental data from similar systems [14].

Paloncyova et al. [57] performed a set of simulations using a multiscale approach similar to Ref. [22], combining coarse-grained MARTINI simulations with atomistic simulations. The Pfizer-Biontech COVID-19 vaccine formulation including, in some case, the PEGylated lipids and long RNA strands (10 to 200 nucleotides of poly-uracile or viral fragments) was considered. The simulated systems contained from 1000 to 4000 lipids, and up to 500 nucleotides in one or more chains. Coarse-grained simulations of self-assembly of a full LNP at low pH, with protonated ILs, showed the formation of an irregular hexagonal phase encapsulating the RNA, as well as the formation of water droplets inside the core of the LNP. In contrast to the works described above, the self-assembly in this case occurred spontaneously in a single step, with core and outer shell forming at the same time. Unlike the rigid siRNA used in Ref. [26] the chains of poly-uracile aggregated to form ring-like supermolecular assemblies. In the absence of RNA, instead, several vesicles formed and aggregated into a single large complex. After assembly at low pH, the authors simulated the exposure of the LNPs to a physiological environment, as in the dilution preceding application or storage of the LNP. This was done by neutralizing all the present ILs of the LNP. The neutralization of the charge leads to a quick expulsion of the RNA or, in some cases, to its encapsulation by inverted micelles made of DSPC. The neutral ILs form a dense phase mixed with cholesterol in the interior of the LNP, while the remaining DSPC migrates to the surface of the LNP.

## LNP mechanisms of action

After reaching the target tissue, the LNPs are believed to enter the cell by endocytosis [20]. Inside the endosome, the LNPs are gradually exposed to decreasing pH. To mimic this phenomenon, Paloncyova et al. [57] took the self-assembled and neutralized LNPs described in the previous section and protonated the ILs. They observed the formation of water vesicles inside the LNP and an associated swelling of the NP. The authors speculate that the observed swelling may represent a possible pathway to endosomal membrane rupture and consequent RNA escape.

**Fig. 8:**
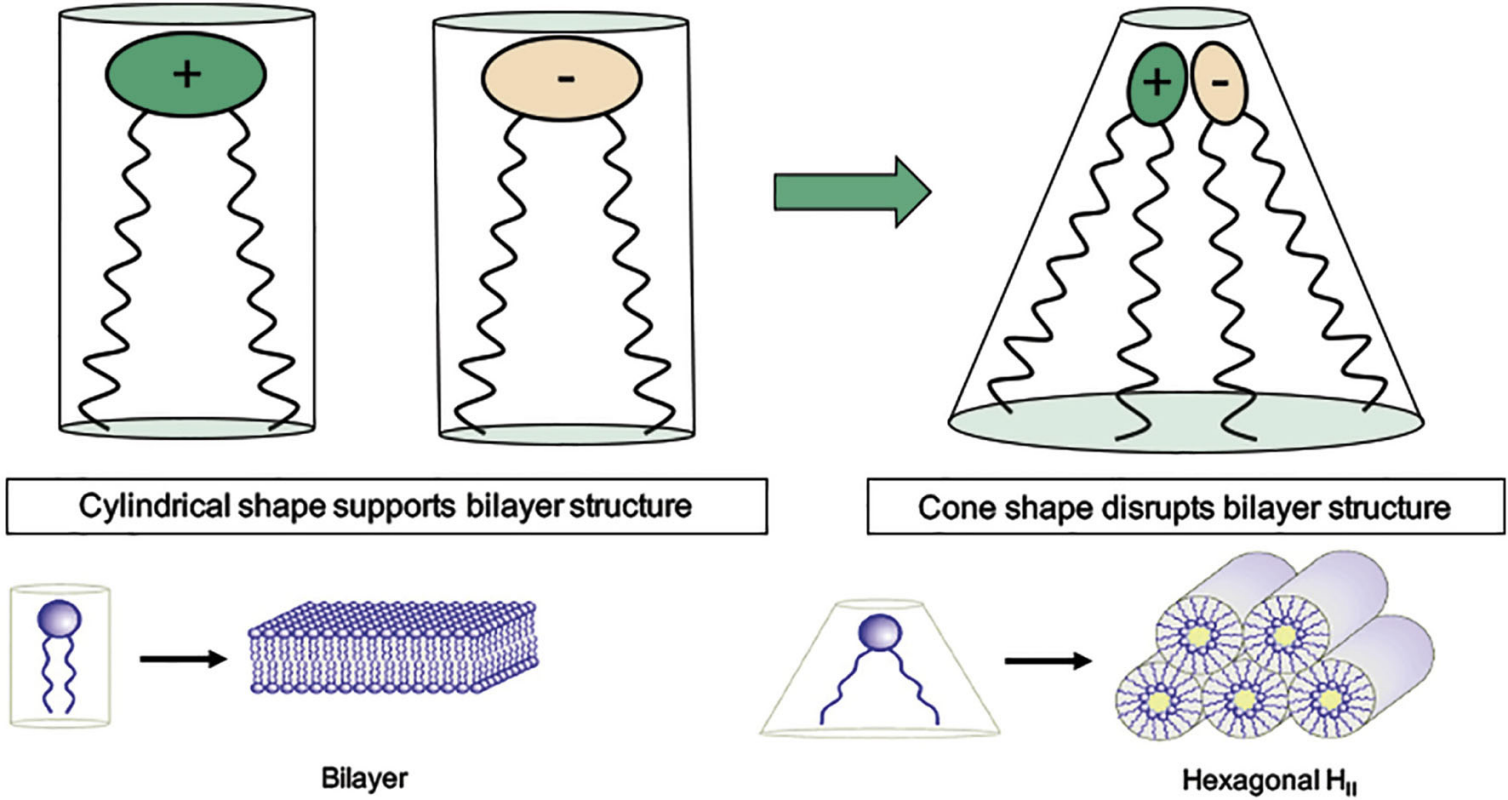
Illustration of the cone-shape hypothesis. While anionic and cationic lipids alone may favor the lamellar phase, that is a bilayer structure (left-hand side of the panel), their association following LNP fusion with endosomal membrane originates a cone-shaped complex which favor the hexagonal phase, thus destabilizing the endosomal membrane. Figure from Ref. [59] Copyright©2017 The American Society of Gene and Cell Therapy under license CC BY-NC-ND 4.0 DEED

The physical rupture of the endosomal membrane by swelling is only one of the possible mechanisms which may lead to RNA escape. Indeed the early hypothesis which led to the use of (ionizable) cationic lipids for nucleic acid delivery, was formulated by Cullis and coworkers [58] after realizing that a mixture of cationic and anionic lipids (the latter being abundant in the endosomal membrane) favors the transition from the lamellar to the inverted hexagonal phase (Fig. 8). Thus, the fusion of a LNP, rich in cationic lipids, with the endosomal membrane, rich in anionic lipids, would destabilize the lamellar state of the endosomal membrane and help forming pores.

**Fig. 9:**
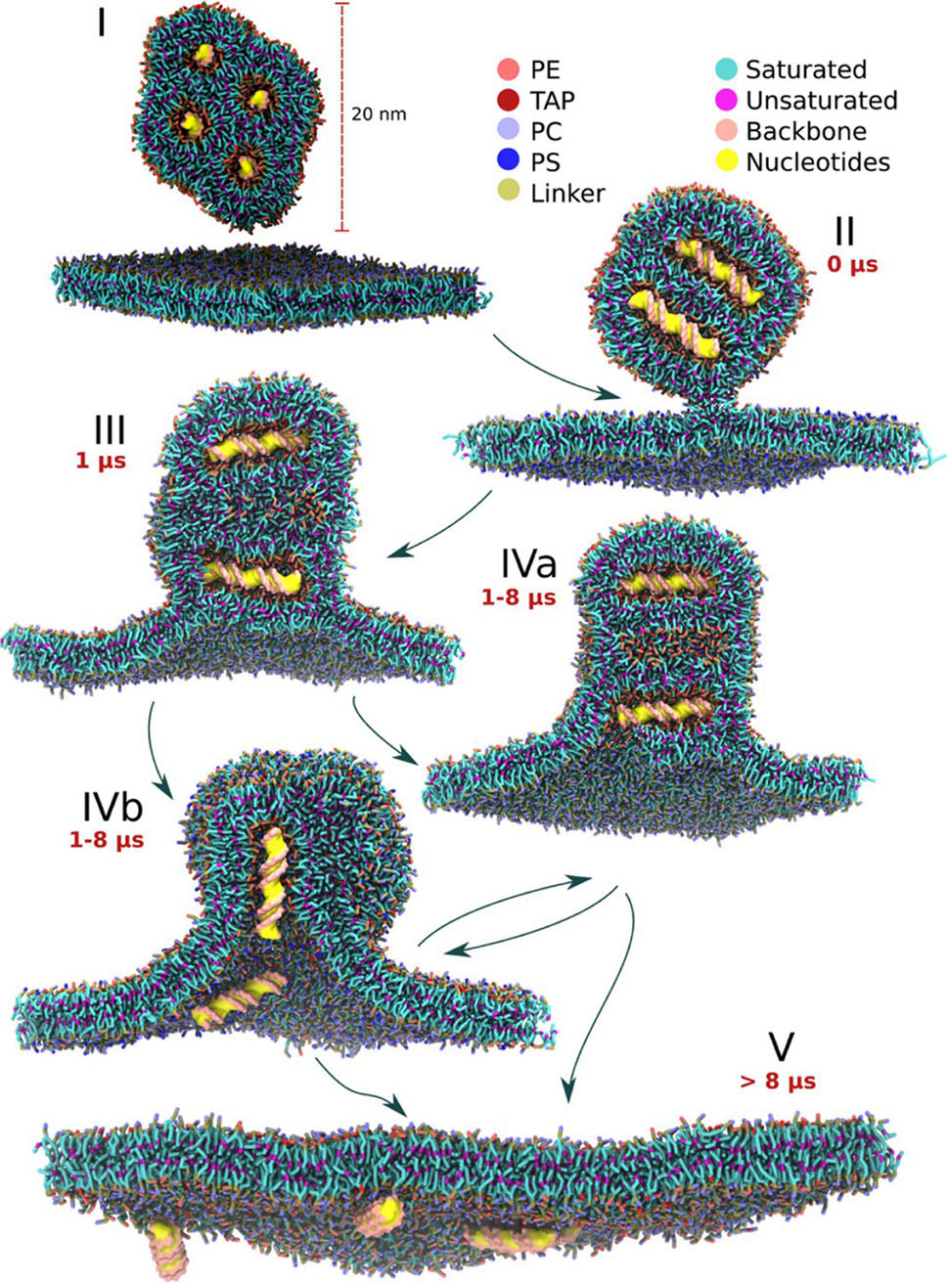
Phases of the simulations of transfection from Ref. [55]. Figure from Ref. [55] copyright©2020 Bruininks et al. under license CC

Bruininks et al. [55] investigated this phenomenon by simulating, using a coarse-grained MARTINI [27, 56] representation, the fusion process between model LNPs, built as described in the same work mentioned in the previous section, and either a segment of a model endosomal membrane or even a model endosomal vesicle. After the formation of a fusion stalk between the LNP and the endosome, which was enforced by an external pulling potential, fusion of the two objects proceeded spontaneously and led in several cases to the release of the dsDNA cargo to the other side of the endosomal membrane (Fig. 9). The release occurred by two main pathways that can be distinguished by the orientation of the dsDNA (parallel or orthogonal) with respect to the membrane at the moment of the formation of the pore. The authors varied the composition of both the LNP and the endosomal membrane and verified that the release of nucleic acid occurred only if the IL in the LNP had unsaturated tails. In addition, it occurred more frequently when the model endosomal membrane contained lipids with short chains or a small degree of unsaturation in the tails. Interestingly the authors pointed out how the endosomal escape is the result of a balance between structure stabilization (unsaturated ILs stabilize the hexagonal phase in the LNP, while saturated ILs destabilize it leading to poorer transfection) and destabilization (ILs with single unsaturation make the hexagonal structure sufficiently stable for transfection, double unsaturation makes the hexagonal phase too stable, and fusion is delayed).

The destabilization of the endosomal membrane due to the mixing of cationic and anionic lipids, which may favor the hexagonal phase over the lamellar, has been explained invoking the cone-shape hypothesis [58], as mentioned earlier. According to it the complex formed by a cationic and an anionic lipid would present an inverted conical shape unlike the two lipids taken separately (Fig. 8). The presence of these complexes in the mixed bilayer would favor the transition to an inverted hexagonal phase, thus leading to endosomal membrane destabilization. Ramezanpour et al. [60] aimed to test the effect of the fusion between the LNP and the endosomal membrane, and in particular the hypothesis that the mixing of cationic lipids with the anionic lipids naturally present in the endosomal membrane may help and destabilize the endosomal bilayer (i.e., in a lamellar phase) in favor of an inverted hexagonal phase (H$_{II}$) leading to endosomal membrane poration or rupture. To this extent they considered several mixtures of the IL KC2, in its protonated form, with cholesterol and the anionic phospholipid DSPS (1,2-Distearoyl-sn-glycero-3-phosphoserine, a lipid with saturated tails), where they varied the relative mol% of each component including water. The systems were set initially in the H$_{II}$ phase, with the water molecules forming a tube along the Z axis surrounded by the lipid heads. The angles between the box vectors of the periodic simulation box were set to stabilize the hexagonal phase. The simulations were run using the CHARMM36 [35–37] force field first for 300 ns at 343 K, to speed up lipid diffusion, and then for another 300 ns at 313 K. The simulations showed that cholesterol tends to co-localize with the saturated lipid DSPS and increases its tail order parameters; the stretched saturated tails of DSPS (with nearby cholesterol) help fill the large volume available at the interstitial space between three tubes of the H$_{II}$ phase. KC2, with its poly-unsaturated (and thus factually shorter), tails weakly localizes in the intertubular region (i.e., in the space between two adjacent tubes). Larger mol% of cholesterol destabilize the H$_{II}$ phase. No evident stabilization due to co-localization of anionic and cationic lipid is observed.

## Conclusion and perspectives

Molecular dynamics simulations have been used to address a large number of questions arising from the experimental characterization of the LNPs as delivery system for nucleic acid therapies. In particular they helped to shed light on the molecular structure of the nanoparticle and how this structure is dependent on LNP composition and environmental conditions.

More specifically, simulations show that at low pH, where electrostatics plays a major role, protonated ILs are generally able to mix with helper lipids (both phospholipids and cholesterol) and preferentially interact with the nucleic acid, while the structure they form, lamellar, or inverted-hexagonal may also depend on the nature and molar fraction of helper lipid present, as well as on the hydration level. Instead, at high pH, some lipid formulations show the tendency to form an oily phase, which includes ILs and, to some extent, cholesterol. This phase is typically surrounded by a leaflet of helper lipids (phospholipids, cholesterol, PEG lipids). The appearance of this phase separation is dependent on the particular IL being considered and its concentration: for example KC2 shows a larger tendency to phase separate than MC3 [41], and in DODMA at low concentration this is not observed [22]. The quantification of the direct interactions between RNA and ILs obtained with simulations has shown relatively weak binding even at low pH [22] and complex correlations with transfection efficiency of lipid formulations [48]. Given the results described above, it is unclear if a medicinal chemistry approach aimed to optimizing the interactions between RNA and IL may be a viable method to improve the efficacy of the formulations. The interactions with the other components of the formulation as well as the emerging collective phenomena (phase separations) may play a major role, which could be missed by focusing on RNA-IL interactions only.

Simulations of LNP self-assembly at low pH converge to the picture of a LNP core where nucleic acids are encapsulated in a disordered inverted-hexagonal or inverted-micellar structure made mostly of ILs but including also helper lipids. The core is surrounded by a leaflet which contains phospholipids but also ILs and cholesterol, where present in the simulation box. It is important to note that building full LNPs with an inner lamellar structure, instead of the disordered hexagonal phase observed in available simulations, would be much more challenging than what achieved so far due to the larger sizes involved, which may reach several hundred nanometers in diameter [16].

While low pH conditions have been explored in most works, the effects of a change of the self-assembled complex to neutral pH have been investigated less frequently. The intrinsic challenge comes from the fact that it is unclear how the pK$_a$ of each IL changes when it interacts with the polyanionic nucleic acid. The expulsion of RNA from the lipid phase observed in several simulation works [47, 57] following complete deprotonation of the ILs, seems to be in contrast with the high encapsulation efficiency observed experimentally, possibly indicating that ILs in the LNPs are not fully neutralized at neutral pH. Presumably, the vicinity of the RNA will lead to an increase of the pK$_a$ of the contacting lipids, which may remain charged even at neutral pH and not undergo a full deprotonation. As mentioned in the previous section, few authors have considered this event by simulating partially deprotonated systems [47], but not on fully assembled LNPs.

Regarding the mechanisms of action of the LNPs, the lack of molecular-level experimental data for cross-validation has so far limited the massive use of molecular dynamics simulations as a tool for investigation. The simulation studies presented above have provided a testing table for experimentally driven hypothesis regarding the mechanism of LNP fusion with the endosomal membrane and the resulting endosomal destabilization. They showed for example how anionic-cationic lipid mixing favors transfection [55] or provided some indication regarding the cone-shape hypothesis [60]. Those studies also helped to make further hypothesis on the precise mechanism of action and how it can be controlled (e.g., the transfection pathways identified in Ref. [55] or the role played by helper lipids in the stabilization of the hexagonal phase in Ref. [60]). These hypothesis are now open for experimental test.

Several questions remain open that could be addressed with molecular dynamics simulations. One important issue regards the role of pH on the structure and mechanism of action of the LNPs. So far, pH has been controlled simply by de/protonating part of the lipids present in the simulation box at the beginning of the simulation. This situation however does not represent the real system, where the protonation state of molecules can change according to the pH of the solution, the pK$_a$ of the molecule and the local electrostatic potential. The availability of new techniques for simulating systems with a large number of ionizable groups at constant pH [61] may pave the way to a more realistic treatment of the ionization state of ILs and help understand more precisely the way they interact with the poly-anionic nucleic acids, both inside the LNP and in the endosome.

Simulations will also play an important role in providing more precise descriptions of the mechanisms of action of the LNPs. Major drawbacks in this direction are represented by the large sizes of the involved systems and the small amount of pertinent experimental data available, which are required to set up and cross-validate the simulations. To address the size problem, coarse-grained models as those already presented in Ref. [55, 57] could represent a possible solution. However, the accuracy of the force fields will need to be carefully tested, and also a careful choice of the system setup will need to be made (e.g., the way LNP are assembled, the way they are put in contact with the endosomal membrane, etc.), which allows for a sensible and unbiased comparison between different systems. Regarding the availability of experimental data, the increasing amount of single-molecule or single-particle experiments in the field is steadily improving our knowledge of those systems. For example single-molecule imaging experiments are being used to localize more precisely some of the components of mRNA therapeutics during the endosomal uptake and escape process [62]. They may help and understand at which stage of the endosomal life cycle the transfection occurs more efficiently. Similarly, surface-sensitive fluorescence microscopy with single LNP resolution is being used to elucidate the pH-dependent mechanism of binding of LNPs on supported endosomal model membranes, demonstrating that large-scale changes to the LNP occur when the pH is lowered from 6 to 5 [63]. These new information could be used to reproduce *in silico* the corresponding conditions and their molecular effect on the LNPs.

Going to large system sizes could be avoided, by focusing on specific experimentally driven hypothesis as, for example, the one related to the lamellar to hexagonal phase equilibrium shift determined by the interactions between the ILs of the LNP and the anionic lipids in the endosomal membrane. Simulations may help understand more clearly how these interactions favor the hexagonal versus the lamellar phase and how the shift in the equilibrium phase may lead to poration or rupture of the endosomal membrane. To this respect, simulations already hinted to a necessary balance between stability and instability of the LNP hexagonal structure [55] to achieve nucleic acid transfection. This topic needs to be explored in more detail, also in light of the fact that recent findings show that the clinically active structure of LNPs represents a transient state of the system [64] and that it is possibly its metastability at the basis of its efficacy.

Ultimately, molecular dynamics simulations represent an invaluable tool to help and understand the structure and mechanism of action of LNPs for nucleic acid delivery and, flanked by well-focused experimental data, both for validation and to drive the models, they may help and discover important relationships between IL chemical structure, LNP morphology and, eventually, their transfection efficiency, thus allowing for a broadening of the range of applications of RNA therapeutics.

## Data Availability

There are no data in this paper to be made available.
